# Melatonin suppresses sympathetic vasomotor tone through enhancing GABA_A_ receptor activity in the hypothalamus

**DOI:** 10.3389/fphys.2023.1166246

**Published:** 2023-03-29

**Authors:** Qiyao Yu, Qi Guo, Sheng Jin, Chao Gao, Peiru Zheng, De-Pei Li, Yuming Wu

**Affiliations:** ^1^ Department of Physiology, Hebei Medical University, Shijiazhuang, China; ^2^ Office of Academic Research, The Fourth Hospital of Hebei Medical University, Shijiazhuang, China; ^3^ Experimental Center for Teaching, Hebei Medical University, Shijiazhuang, China; ^4^ Department of Radiation Oncology, The Fourth Hospital of Hebei Medical University, Shijiazhuang, China; ^5^ Department of Medicine, University of Missouri, Columbia, KY, United States; ^6^ Hebei Collaborative Innovation Center for Cardio-Cerebrovascular Disease, Shijiazhuang, China

**Keywords:** autonomic nervous system, circadian rhythms, hypothalamus, melatonin, sympathetic nervous system, GABA_A_ receptor

## Abstract

**Introduction:** Melatonin (5-methoxy-N-acetyl-tryptamine) is a circadian hormone synthesized and secreted by the pineal gland. In addition to regulating circadian rhythms of many physiological functions, melatonin is involved in regulating autonomic nervous function and blood pressure. Hypothalamus paraventricular nucleus (PVN), receiving melatonin projections from the superchiasmatic nucleus, is a critical brain region to regulate neuroendocrine and cardiovascular function. Here, we determined the synaptic mechanisms involved in the effect of melatonin on the sympathetic outflow and blood pressure.

**Methods and Results:** Microinjection of melatonin into the PVN produced a depressor effect and decreased renal sympathetic nerve activity (RSNA). While microinjection of luzindole, a non-selective melatonin receptor antagonist, into the PVN did not change melatonin-induced sympathoinhibition, GABA_A_ receptor antagonist bicuculline eliminated melatonin-induced sympathoinhibition. Furthermore, melatonin decreased firing rate of retrogradely labeled PVN neurons which project to the rostral ventrolateral medulla (RVLM), an effect was not altered by luzindole but eliminated by bicuculline. Melatonin significantly increased the amplitude of spontaneous and evoked GABAergic inhibitory synaptic currents, as well as GABA-induced currents.

**Conclusion:** These data suggest that melatonin in the PVN suppresses sympathetic vasomotor tone through enhancing GABA_A_ receptor activity. This study provides novel information for understanding the cellular mechanisms involved in the effect of melatonin on regulating blood pressure and sympathetic output.

## 1 Introduction

Melatonin is a natural compound found in different organisms including bacteria, and later identified as a hormone synthesized in darkness by the pineal gland of mammalian (Mannino et al., 2021). Pineal gland release melatonin into the circulation and the cerebrospinal fluid to be involved in regulating sleep–wake timing and blood pressure regulation, and in control of seasonal rhythmicity of physiological activities (Arangino et al., 1999; Tricoire et al., 2003). Melatonin synthesis and secretion are regulated by the circadian pacemaker suprachiasmatic nucleus (SCN) at the hypothalamus, which receives input from photosensitive ganglion cells in the retina through the optic nerve (Reppert and Weaver, 2002; Herzog, 2007). Previous studies have shown that pinealectomy is associated with an increase in blood pressure in normotensive rats (Zanoboni and Zanoboni-Muciaccia, 1967; Karppanen et al., 1975; Vaughan et al., 1979; Kurcer et al., 2006), while administration of melatonin produces an depressor response in spontaneously hypertensive rats (Kawashima et al., 1987; Girouard et al., 2001; Pechanova et al., 2007) and essential hypertension patient (Jonas et al., 2003; Scheer et al., 2004; Simko and Paulis, 2007). Both peripheral mechanisms and central mechanism of the antihypertensive effects of melatonin are proposed (Chuang et al., 1993; Reiter et al., 2005; Simko and Paulis, 2007). Melatonin induced depressor response appears to be associated with an inhibition of basal sympathoadrenal tone (Laflamme et al., 1998; Scheer et al., 2003) and improved baroreflex responses (Girouard et al., 2004). Furthermore, microinjection of melatonin into the anterior hypothalamic area produced a dose-related decrease in arterial pressure in rats with stress-induced hypertension (Ding et al., 2001). Previous studies have revealed a receptor-independent action of melatonin, including a direct interaction with calmodulin (Turjanski et al., 2004), inhibition of Ca^2+^ channels (Satake et al., 1986), activation of GABA-receptors (Wang et al., 2003), reduction of oxidative stress (Galano, 2011; Galano et al., 2011; Zhang and Zhang, 2014), and inhibition of nitric oxide (NO) signaling (Aydogan et al., 2006). Thus, the mechanisms involved in central effect of melatonin on regulating blood pressure remains to be elucidated.

Neurons in the paraventricular nucleus (PVN) of the hypothalamus provide excitatory drive to sympathetic outflow through direct projection to the preganglionic neurons in the intermediolateral cell column in spinal cord and the rostral ventrolateral medulla (RVLM), which innervates generates sympathetic tone and regulating arterial pressure (Coote et al., 1998; Pyner and Coote, 2000; Cano et al., 2001; Cano et al., 2004; Li et al., 2017). It has been shown that infusion of melatonin into the PVN attenuates myocardial injury during ischemia–reperfusion through inhibition oxidative stress and inflammatory cytokines (Yang et al., 2019). Excitatory output of PVN presympathetic neurons is intermittently inhibited by GABAergic innervation from neurons in suprachiasmatic nucleus (SCN) (Kalsbeek et al., 2000; Cui et al., 2001), which is a key brain region regulating circadian rhythm in sympathetic vasomotor tone (Buijs et al., 1999; Scheer et al., 2003). It has been shown that melatonin enhances GABA_A_ receptor activity (Wan et al., 1999; Wang et al., 2003; Naguib et al., 2007). Therefore, melatonin may inhibit sympathetic tone through enhancing GABAergic signals from the SCN (Kalsbeek et al., 2000; Cui et al., 2001). In this study, we used RVLM projecting PVN neurons to determine the cellular mechanisms involved in the depressor effect of melatonin in the PVN.

Previous studies have shown that melatonin enhances GABA binding in the rat brain (Coloma and Niles, 1988; Niles, 1991). Melatonin potentiated GABA-evoked current amplitude through direct interaction with GABA_A_ receptors in SCN neurons which express melatonin receptor 1 (MT1) (Wan et al., 1999). It has been shown that MT1 melatonin receptors are distributed in the hypothalamus including the PVN, and expressed on neurons synthesizing vasopressin, oxytocin, or corticotropin releasing hormone (Wu et al., 2006). It has been shown that melatonin acts as a positive allosteric modulator of GABA_A_ receptors since the effect of melatonin on GABA_A_ receptors was not blocked by antagonizing melatonin receptors with luzindole (Li et al., 2001). In this study, we directly examined the effect of melatonin on RVLM projecting PVN neurons and assessed its potential mechanisms.

## 2 Methods

### 2.1 Animals

Adult (12–15 weeks old) male Sprague Dawley (SD) rats were used in this study. These rats were housed at an ambient temperature with free access to food and water *ad libitum* in a 12-h light/dark cycle. The experimental protocol (#20220105) was reviewed and approved by Animal Care and Use Ethics Committee of the Fourth Hospital of Hebei Medical University. The protocols and procedures performed were in compliance with the Guide for the Care and Use of Laboratory Animals (NIH Publication No. 85–23, revised 2011). All efforts were made to minimize animal suffering, to reduce the number of animals used, and to utilize alternatives to *in vivo* techniques.

### 2.2 *In Vivo* recordings of hemodynamics and renal sympathetic nerve activity

In this procedure, SD rats were anesthetized by a mixture of urethane (800 mg/kg) and α-chloralose (60–75 mg/kg, intraperitoneal injection) and ventilated mechanically with pure oxygen using a rodent ventilator through a trachea cannulation. The CO_2_ concentration in exhale was monitored by a CO_2_ analyzer and maintained at 4%–5% by adjusting the ventilation rate at 50–70 breaths per minute, and the tidal volume of 2–3 mL. The pulsatile arterial blood pressure (ABP) was recorded by a catheter inserted into the right-side femoral artery. Heart rate (HR) was calculated based on the pulsatile pressure signal. After exposure the postpositional cavities on the left side, the renal sympathetic nerve was exposed, and a branch of renal postganglionic sympathetic nerve was surgically isolated and put on a recording electrode to record the nerve discharge. The nerve discharge electrical signals were amplified, bandpass filtered (100–3,000 Hz) using a high-impedance differential amplifier (AM3000H, AM system), and monitored by the software screen and an audio amplifier. The renal sympathetic nerve activity (RSNA) and ABP were digitized at 10 kHz by analog-to-digital interface and recorded by LabChart Pro (AD Instruments). At the end of recording, the background noise of recording of renal sympathetic nerve was determined by completely suppressing renal sympathetic nerve activity by elevation of ABP elicited by a bolus intravenous injection of phenylephrine (4 μg/kg). The change of RSNA percentage from the baseline was calculated and presented.

### 2.3 Microinjection into the paraventricular nucleus

For the PVN microinjections, with the rat heads fixed on a stereotactic apparatus. A burr hole was drilled on the skull following coordinates microinjection into the PVN: 1.6–2.0 mm caudal to the bregma, 0.5 mm lateral to the midline, and 7.0–7.5 mm ventral to the dura (Li et al., 2017). The solution containing the agents in a volume of 100 nL for each injection were microinjected into the PVN bilaterally through a glass microinjection pipette with a tip diameter of 20–30 μm connected to a calibrated microinjection system (Nanojector III, Drummond Scientific Company, Broomall, PA). The injection solution contained 1% FluoSpheres microspheres (0.04 μm; Invitrogen, Eugene, OR) to estimate the injection site and drug diffusion size. The rat brain was removed and fixed in 4% paraformaldehyde solution after completing each experiment. Frozen coronal sections (50-μm thick) were cut at the PVN level and were viewed by a microscope for fluoSpheres fluorescent regions. The data from this rat were excluded from analysis if the microinjection site was outside of the PVN.

### 2.4 Retrogradely labeling of rostral ventrolateral medulla projecting paraventricular nucleus neurons

The RVLM projecting PVN neurons were retrogradely labeled by FluoSpheres, as described previously (Li et al., 2003; Li et al., 2017). Briefly, the rats were anesthetized with 2%–3% isoflurane. Burr holes (about 4 mm in diameter) were made bilateral by a microdrill in the occipital bone following coordinates (bregma): 12.0-13.0-mm caudal, 1.9-2.2-mm lateral, and 7.9-8.1-mm deep from the dura. A rhodamine-labeled fluorescent microsphere suspension (FluoSpheres, 50 nL, 0.04 μm; Invitrogen) was ejected by a microinjector, Nanojector III; Drummond Scientific Company, into the bilateral RVLM. The wound was closed after injection, and the rats were returned to their cages for 1–2 weeks, a period of time that allows the Fluospheres to be transported to the PVN. The rats were treated prophylactically with an analgesic (0.5 mg/kg buprenorphine, s.c., every 12 h for 2 days) and an antibiotic (5 mg/kg enrofloxacin, s.c., daily for 3 days).

### 2.5 *In Vitro* electrophysiological recordings in brain slices

Acute live hypothalamic slices were sectioned from the brain tissue of FluoSphere-injected rats, as described previously (Li et al., 2002; Li et al., 2017). Briefly, under anesthesia with 4%–5% isoflurane, the rats were decapitated, and the brain was quickly obtained and placed in ice-cold artificial cerebrospinal fluid (aCSF) containing the following components (in mM): 124.0 NaCl, 3.0 KCl, 1.3 MgSO_4_, 2.4 CaCl_2_, 1.4 NaH_2_PO_4_, 10.0 glucose, and 26.0 NaHCO_3_, saturated with mixed gas of 95% O_2_ and 5% CO_2_. A tissue block containing at the PVN level was trimmed and fixed on the sectioning stage of a vibrating microtome with tissue glue (VT1200, Leica). Coronal hypothalamic slices in a thickness of 300 μm were cut and then transferred to a incubation chamber containing aCSF continuously gassed with mixed gas of 95% O_2_ and 5% CO_2,_ at a temperature of 34°C for 1 h before recording.

Whole-cell patch-clamp recordings of FluoSphere-labeled PVN neurons were performed in the acutely prepared hypothalamic slices. A brain slice containing the PVN was placed in the recording chamber containing aCSF saturated by 95% O_2_ and 5% CO_2_ and continuously perfused at a speed of 3 mL/min at 34°C maintained by using an inline solution heater. The labeled PVN neurons were initially identified under an upright microscope equipped with epifluorescence illumination and differential interference contrast optics. The recording electrodes were pulled by a micropipette puller from borosilicate capillaries. The resistance of the electrode was 3–6 MΩ when it was filled with an internal solution containing the following components (mM) 140.0 K gluconate, 2.0 MgCl_2_, 0.1 CaCl_2_, 10.0 HEPES, 1.1 EGTA, 0.3 Na_2_-GTP, and 2.0 Na_2_-ATP adjusted to pH 7.25 with 1 M KOH, 270–290 mOsm. Evoked inhibitory postsynaptic current (IPSCs) were recorded at a holding potential of 0 mV and in the presence of 20 μM 6-cyano-7-nitroquinoxaline-2,3-dione (CNQX) and 50 μM AP5 to block ionotropic glutamate receptors. GABA-induced currents were recorded in voltage-clamp mode by puff-application of GABA in a concentration of 100 µM. The miniature IPSCs (mIPSCs) were recorded in the presence of 20 μM CNQX and, 1.0 μM tetrodotoxin (TTX) at a holding potential of 0 mV. Evoked excitatory postsynaptic current (EPSCs) were recorded at a holding potential of −60 mV in the presence of 20 μM bicuculline to blocking GABA_A_ receptors. The miniature EPSCs (mEPSCs) were recorded in the presence of 1 μM TTX and 20 μM bicuculline at a holding potential of −60 mV. The electrical signals were processed using the Multiclamp 700 B amplifier and Digidata 1320A. The recording was abandoned if the input resistance changed more than 15% during the recording. The readings were obtained and averaged during a 3- to 5-min recording period before and after drug application.

### 2.6 Agents

Melatonin, GABA, and luzindole were purchased from Sigma Aldrich (St Louis, MO, United States). Melatonin was dissolved in 0.1% ethanol in normal saline (pH 7.0). These drugs, or 0.1% ethanol in normal saline (vehicle; as a control), were microinjected into the PVN in a volume of 100 nL over a period of 10 s (−)-Bicuculline methochloride and CNQX were obtained from HelloBio. Inc.

### 2.7 Statistical analysis

Data are presented as means ± SEM. The rats were assigned into experimental groups based on randomization numbers generated by Prism 8 (GraphPad Software Inc., La Jolla, CA, United States). Rats or their tissues were coded for the duration of the study, and codes were revealed for identification during data analysis. Quantitative data were analyzed by whom blinded to group-identifying information. The *in vivo* experiments were performed by an investigator blinded to treatment conditions. For electrophysiological recording, one neuron was recorded from one brain slice and at least 3 rats were included in data analysis in each group. The RSNA, ABP, and HR were analyzed using LabChart Pro software. RSNA was integrated off-line after subtracting the background electrical noise. Baseline values of RSNA were obtained by averaging the signal over a 30–60 s period immediately before each treatment. Response values were determined by averaging signals over 30-s period during the maximal responses occurred after each intervention. Spontaneous firing activity, membrane potentials, and evoked currents, were analyzed by using Clampex 11.0 software package. The junction potential was corrected based on various different potentials measured between internal and external solutions. A two-tail Student t test was used to compare two groups, and two-way or one-way ANOVA followed by Dunnett’s *post hoc* test was used to compare means of more than two groups. Statistical analyses were performed by Prism 8 software (GraphPad). *p* < 0.05 was considered statistically significant.

## 3 Results

### 3.1 Microinjection of melatonin into the paraventricular nucleus decreased arterial blood pressure, renal sympathetic nerve activity, and HR

**FIGURE 1:**
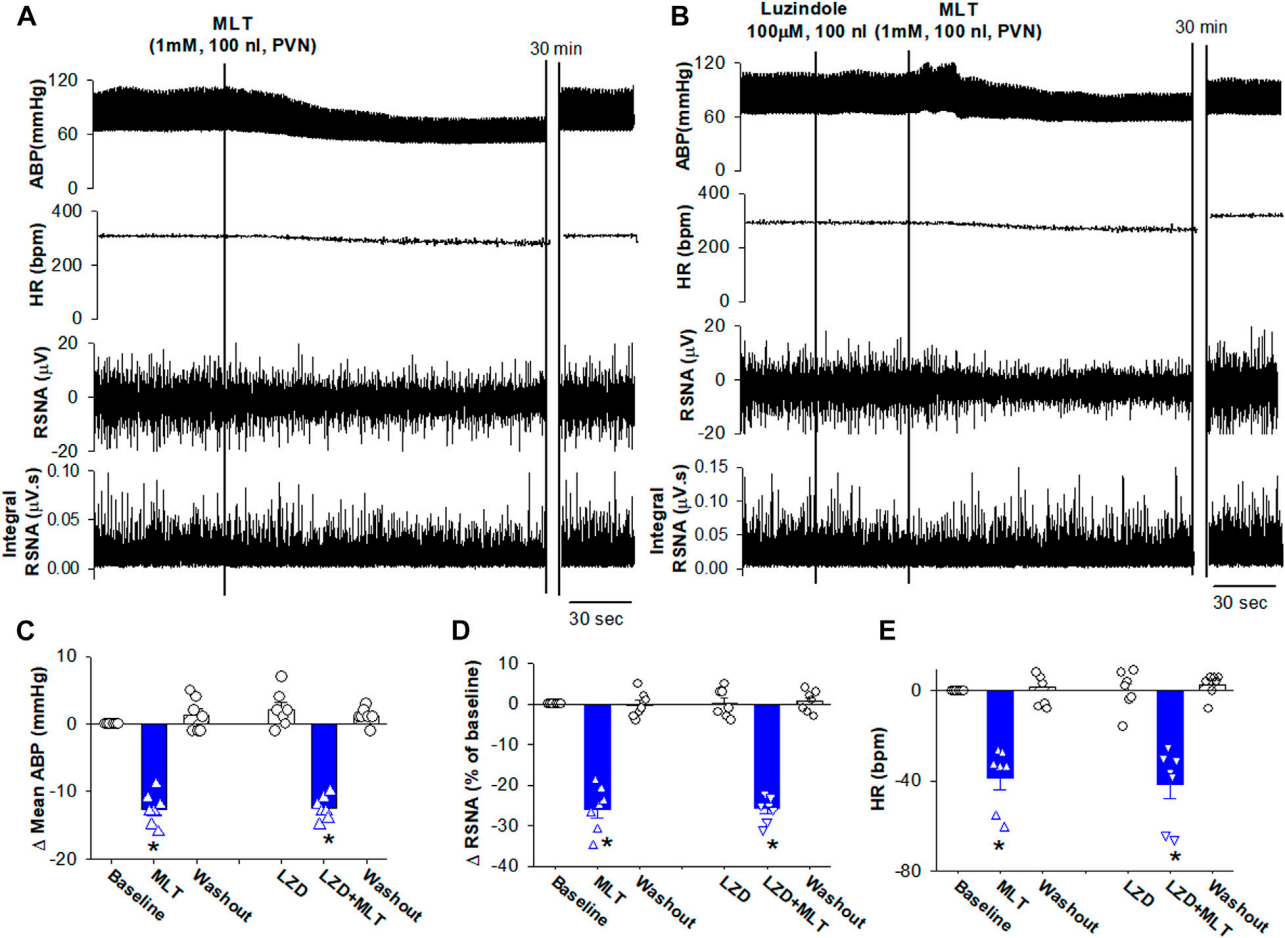
Microinjection of melatonin into the PVN reduced ABP and sympathetic outflow. **(A)**, Representative recordings show that the effect of bilateral microinjection of melatonin (0.1 mM, 100 nL) into the PVN on mean ABP, RSNA, HR, and integrated RSNA (Int-RSNA). **(B)**, Representative recordings show microinjection of non-selective antagonist luzindole (10 mM, 100 nL) failed to alter the melatonin-induced reduction of ABP and RSNA. **(C–E)**, Summary data show change of mean ABP, RSNA, and HR in response to microinjection of melatonin and luzindole, an antagonist for MT1 and MT2 receptors, into the PVN. Data are mean ± SEM. One-way ANOVA with the Bonferroni’s *post hoc* test was used to determine differences between groups (n = 7 rats in each group). *p < 0.05, compared with the respective baseline each group. MLT, melatonin; LZD, luzindole.

We first determined the effect of melatonin in the PVN on controlling sympathetic vasomotor tone Microinjection of melatonin (1 mM, 100 nL) bilaterally into the PVN significantly decreased the mean ABP from 85 ± 1.6 to 72.3 ± 1.7 mmHg (n = 7 rats, *p* < 0.0001, F_(2,18)_ = 95.0), RSNA from 100% to 74% ± 2.1% (*p* < 0.0001, F_(2,18)_ = 131.3), and HR from 302 ± 7.7 to 263 ± 4.7 bpm (*p* < 0.0001, F_(2,18)_ = 59.45; Figures 1A,C–E). The RSNA and mean ABP started to decrease at a mean time of 2.1 ± 0.2 min after injection, and this effect lasted for 16.3 ± 3.5 min. Furthermore, we determine whether melatonin-induced depressor response and sympathoinhibition were mediated by its receptors MT1 or MT2 by using a non-selective antagonist luzindole. Microinjection of luzindole (10 mM, 100 nL) into the PVN did not alter the baseline ABP, RSNA, and HR. Subsequent microinjection of melatonin into the PVN induced a similar depressor response and sympathoinhibition (Figures 1B–E). These data suggest that MT1 or MT2 receptors play minor role in mediating melatonin-induced depressor and sympathoinhibition.

### 3.2 Melatonin decreased firing activity of rostral ventrolateral medulla projecting paraventricular nucleus neurons

**FIGURE 2:**
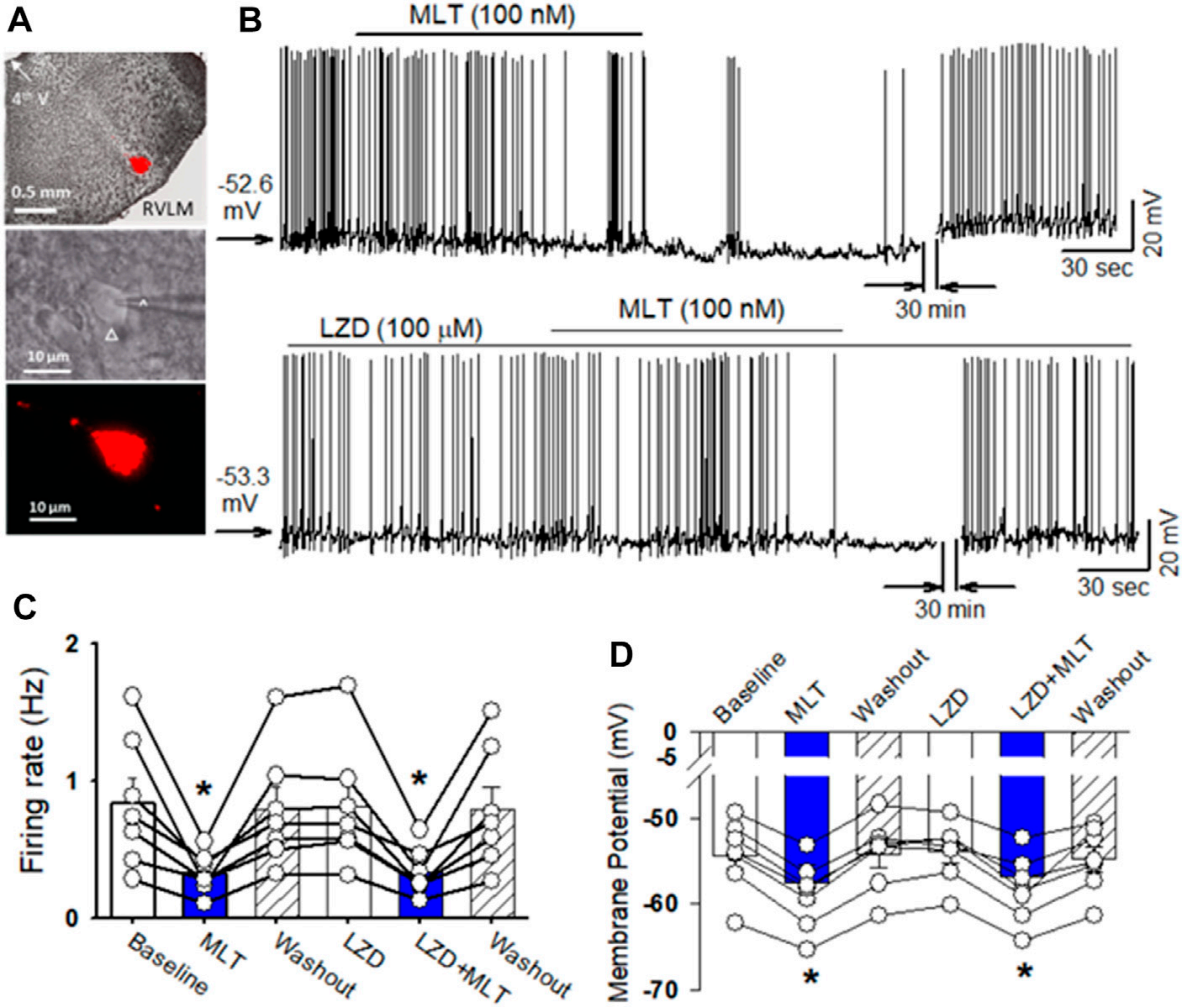
Melatonin reduced the firing activity of RVLM projecting PVN neurons. **(A)**, microphotography (upper panel) and FluoSphere-labeled PVN neuron (*) in brain slice viewed with and infrared differential interference contrast optics (middle panel) and fluorescence illumination (lower panel). The recording electrode was marked with an arrow. **(B)**, upper panel: Representative raw tracings show that bath application of melatonin (100 nM) decreased the firing activity of RVLM projecting PVN neurons. Lower panel: MT receptor antagonist luzindole (100 μM) did not alter the baseline firing activity and melatonin-induced decrease in the firing activity of RVLM projecting PVN neurons. **(C)** and **(D)**, Summary data of firing rats **(C)** and membrane potential **(D)** show melatonin decreased the spontaneous firing rats and hyperpolarized the labeled RVLM projecting PVN neurons in both vehicle and MT receptor antagonist luzindole (100 μM) (n = 7 neurons from 3 rats in each group). Data are mean ± SEM. One-way ANOVA with the Bonferroni’s *post hoc* test was used to determine differences between groups *p < 0.05 compared with the basal values in each group. MLT, melatonin; LZD, luzindole.

To determine the cellular mechanisms underlying melatonin-induced depressor response and sympathoinhibition, the effect of melatonin on the firing activity of PVN neurons that project to the RVLM. In brain slice preparation (Figure 2A). Bath application of melatonin (100 nM) significantly decreased the firing rate of retrogradely labeled RVLM projecting PVN neurons from 0.84 ± 0.2 to 0.32 ± 0.1 Hz (*p* = 0.0098, F_(2,18)_ = 12.54, n = 7 neuron from 3 rats, Figures 2B,C). Furthermore, melatonin (100 nM) hyperpolarized these neurons from −54.3 ± 1.6 to −58.9 ± 1.5 mV (*p* < 0.0001, F_(2,18)_ = 112, Figure 2D). In addition, we tested if melatonin-induced decrease in the firing activity of RVLM projecting PVN neurons was mediated by MT1 or MT2. While bath application of MT1 and MT2 non-selective antagonist luzindole (100 μM) did not alter the basal firing activity. In the presence of luzindole, melatonin still decreased the firing rate of RVLM projecting PVN neurons from 0.8 ± 0.2 to 0.3 ± 0.1 Hz (*p* = 0.004, F_(2,18)_ = 14.5, n = 7 neuron from 3 rats, Figures 2B–D). These data suggest that melatonin decreased the activity of PVN neurons through mechanism independent of MT1 and MT2.

### 3.3 Melatonin enhanced GABA_A_ receptor activity

**FIGURE 3:**
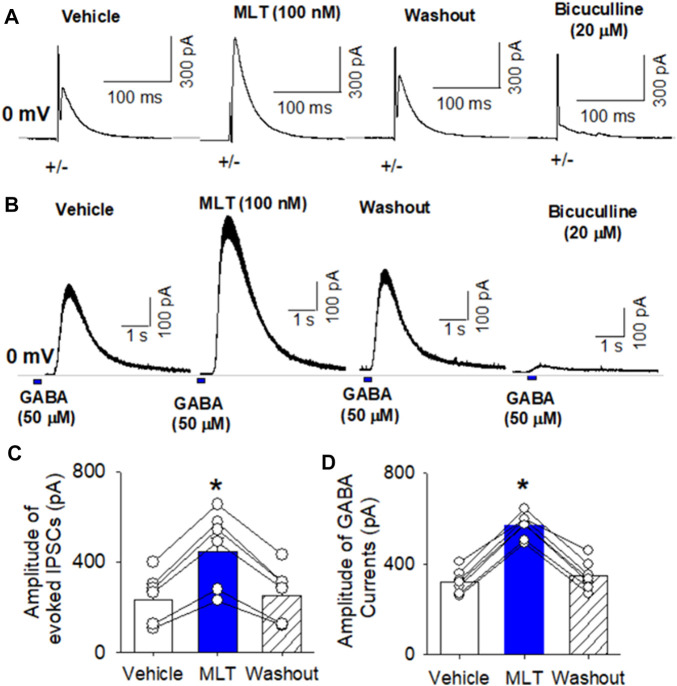
Effect of melatonin on GABAA receptor activity in RVLM projecting PVN neurons. **(A,B)**, Representative raw tracings show that melatonin at concentration of 100 nM increased the amplitude of evoked GABAergic IPSCs **(A)** and the currents induced by puff application of 100 μM GABA **(B)** onto the labeled RVLM projecting PVN neurons recorded. Please note that application of 20 μM bicuculline abolished evoked IPSCs and puff GABA-induced currents. **(C,D)**, Summary data show that 100 nM melatonin significantly increased the amplitude of evoked IPSCs (n = 6 neurons from 3 rats) and puff GABA-induced currents (n = 6 neurons from 3 rats). Data are mean ± SEM. One-way ANOVA with the Bonferroni’s *post hoc* test was used to determine differences between groups *p < 0.05 compared with the basal values in each group. MLT, melatonin.

It has been shown that melatonin directly interact with GABA_A_ receptors (Naguib et al., 2007). To determine if melatonin alters GABAA receptor activity in the PVN neurons, we recorded evoked GABAergic IPSCs and GABA current elicited by puff application of GABA in brain slice preparation using whole-cell recording. Electrical stimulation evoked IPSCs at a holding potential of 0 mV in labeled PVN neurons. Bath application of 100 nM melatonin significantly increased the amplitude of evoked IPSCs from 234.6 ± 40 to 445.1 ± 60 pA (*p* < 0.0001, F_(2,18)_ = 89.7, n = 7 neuron from 3 rats, Figures 3A,C). Furthermore, puff GABA (100 μM) elicited outward currents at a holding potential of 0 mV (Figure 3B). Bath application 100 nM melatonin significantly increased the GABA-induced currents from 321.5 ± 19.6 to 568.9 ± 20.6 pA in 8 labeled PVN neuron from 3 rats (*p* < 0.0001, F_(2,21)_ = 263.5, Figures 3B,D). Application of bicuculline (20 µM) abolished evoked IPSCs and GABA-induced currents (Figures 3A,B).

**FIGURE 4:**
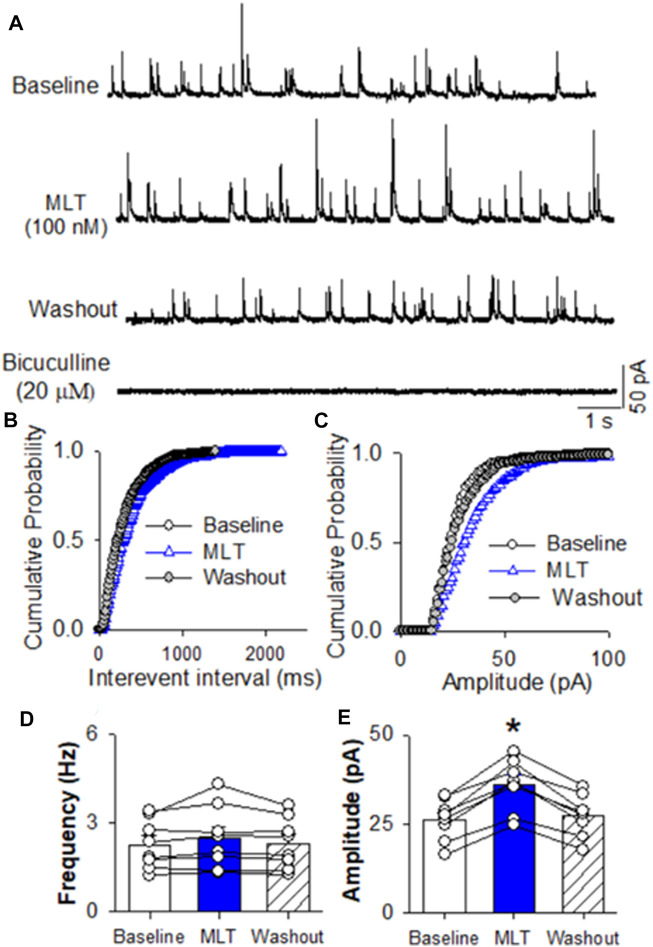
Effect of melatonin on GABAergic miniature IPSCs in RVLM projecting PVN neurons **(A)** Representative tracings from a labeled RVLM projecting PVN neuron show that mIPSCs recorded during control, application of 100 nM melatonin, washout, and application of 20 μM bicuculline. Note that bicuculline completely eliminated mIPSCs. **(B,C)**, Cumulative probability plot analysis of mIPSCs of the same neuron showing the distribution of the interevent interval **(B)** and peak amplitude **(C)** during control, melatonin application, and washout. Melatonin shifted the distribution curve of amplitude of mIPSCs to the right (*p* < 0.05; Kolmgorov—Smirnov test) without changing the distribution of the interevent-interval. **(D,E)**, Summary data show the effect of 100 nM melatonin on the frequency **(D)** and amplitude **(E)** of mIPSCs of 8 labeled PVN neurons. Data are presented as means ± SEM (*p < 0.05 compared with the control; Kruskal–Wallis ANOVA, followed by Dunn’s *post hoc* test). MLT, melatonin.

**FIGURE 5:**
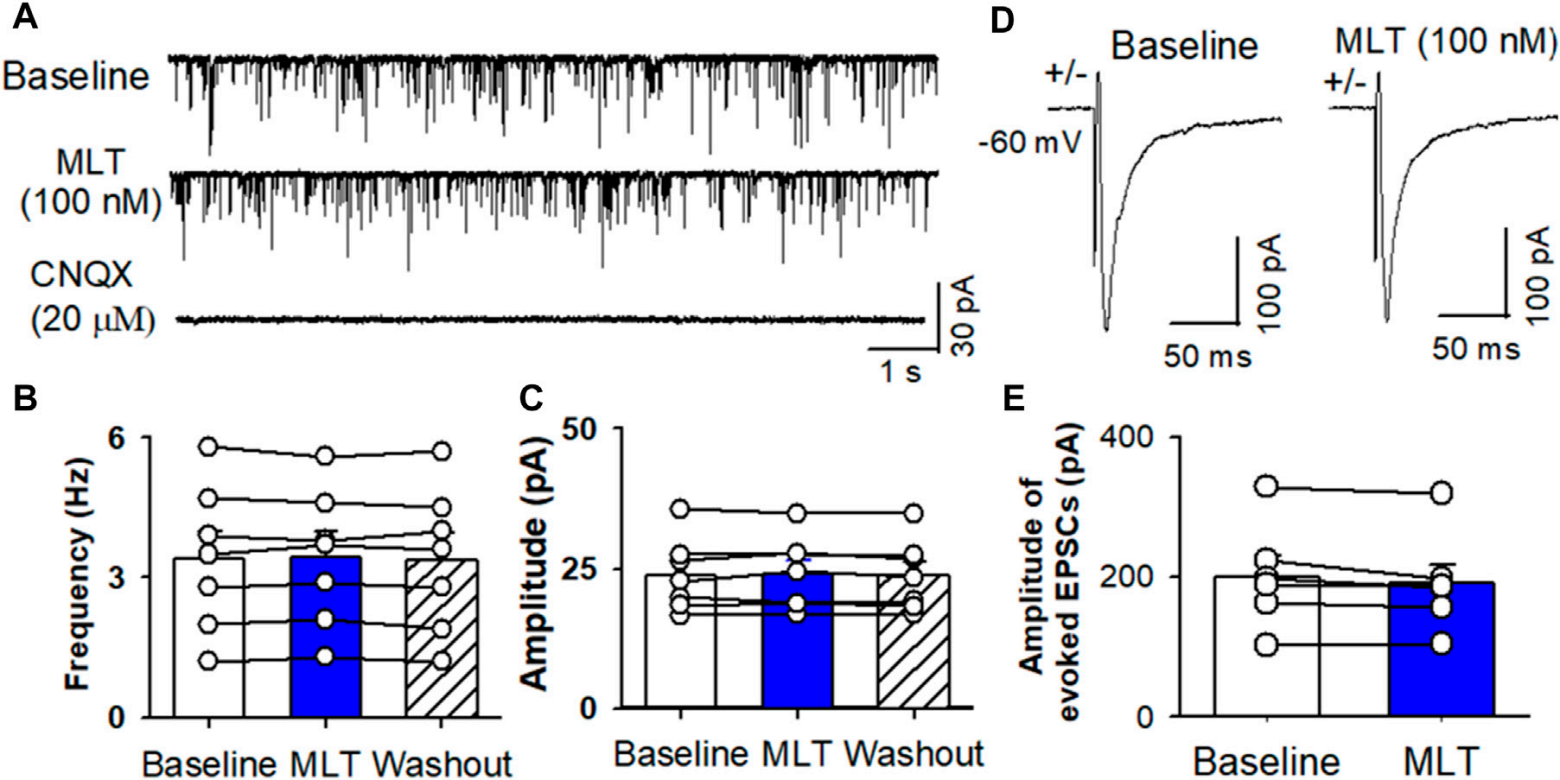
Effect of melatonin on glutamatergic mEPSCs and evoked EPSCs in RVLM projecting PVN neurons. **(A)** Representative raw tracings from a labeled RVLM projecting PVN neuron show that mEPSCs recorded during control, application of 100 nM melatonin, and application of 20 μM CNQX. **(B,C)**, Summary data show the effect of 100 nM melatonin on the frequency **(B)** and amplitude **(C)** of mEPSCs of 6 labeled PVN neurons. **(D,E)**, Representative raw tracings **(D)** and summary data of amplitude of evoked EPSCs **(E)** show that 100 nM melatonin did not change the amplitude of evoked EPSCs. Data are presented as means ± SEM (*p < 0.05 compared with the control; ANOVA, followed by Dunn’s *post hoc* test). MLT, melatonin.

To determine if melatonin has presynaptic effect, we assessed the miniature IPSCs in labeled PVN neurons. The miniature IPSCs were recorded in the presence of 1 μM TTX and 20 μM CNQX. Application of 20 μM bicuculline abolished mIPSCs (n = 8) (Figure 4A). Application of melatonin in a concentration of 100 nM significantly increased the amplitude from 26.0 ± 2.1 to 35.8 ± 2.7 pA (*p* < 0.0001, F_(2,21)_ = 68.11, n = 8 neurons from 3 rats, Figures 4D,E), without affecting the frequency of the mIPSCs in 8 neurons tested. The cumulative probability analysis of mIPSCs before and during melatonin application revealed that the distribution pattern of the interevent interval of mIPSCs was not altered, whereas the distribution pattern of the amplitude was shifted to the right (Figures 4B,C). However, application of 100 nM melatonin did not alter the frequency and amplitude of miniature EPSCs, which were recorded in the presence of 20 μM bicuculline at a holding potential of −70 mV (Figures 5A–C). In addition, melatonin (100 nM) did not change the amplitude of evoked EPSCs in RVLM projecting PVN neurons (Figures 5D,E).

### 3.4 Blocking GABA_A_ receptors eliminated melatonin-induced decreases in the firing activity of rostral ventrolateral medulla projecting paraventricular nucleus neurons and sympathoinhibition

**FIGURE 6:**
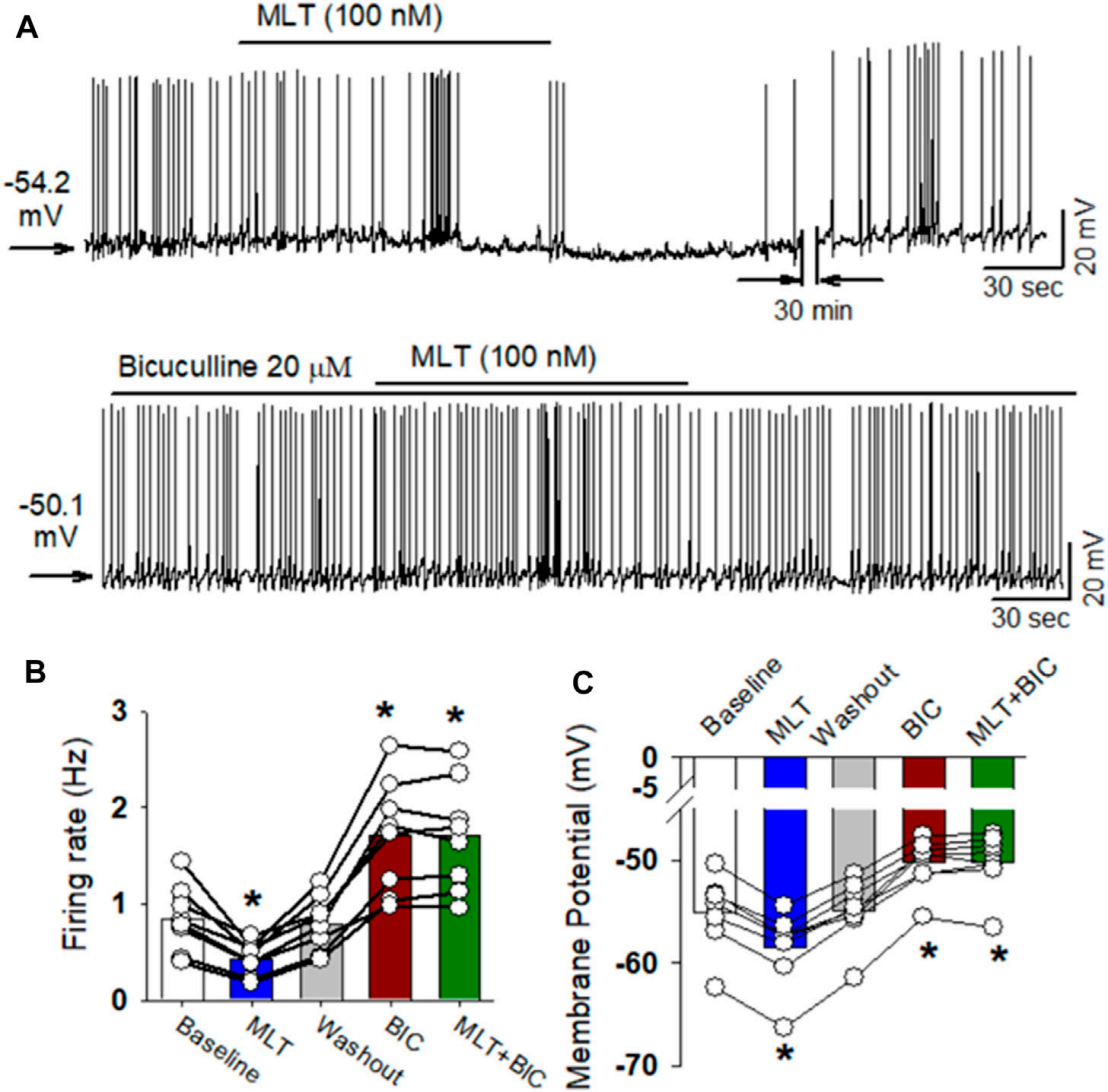
GABAA receptor antagonist eliminated melatonin-induced decrease in firing activity of RVLM projecting PVN neurons. **(A)**, Representative raw tracings showing the spontaneous firing activity during application of 100 nM melatonin, 20 μM bicuculline, bicuculline plus melatonin. Please note that application of bicuculline significantly increased the firing activity of RVLM projecting PVN neurons. Furthermore, bicuculline eliminated the effect of melatonin on the firing activity and membrane potentials. **(B,C)**, summary data of firing rate **(B)** and membrane potentials **(C)** showing that GABA_A_ receptor antagonist bicuculline eliminated melatonin-induced decrease in firing activity and hyperonization of RVLM projecting PVN neurons. Please note that bicuculline increased the firing activity and depolarized the membrane potentials of RVLM projecting PVN neurons. Data are presented as means ± SEM (*p < 0.05 compared with the control; ANOVA, followed by Dunn’s *post hoc* test). MLT, melatonin.

Since melatonin preferentially enhanced GABA_A_ receptor activity in the PVN neurons, we speculate that GABA_A_ receptors mediate the inhibitory effect of melatonin on labeled PVN neurons. To test this hypothesis, the effect of 100 nM melatonin on spontaneous firing activity of labeled PVN neurons in the presence of GABA_A_ receptor antagonist bicuculline. Application of bicuculline at a concentration of 20 μM significantly increased the firing rate of 7 labeled PVN neurons. Subsequent application of 100 nM melatonin did not significantly change the firing rate in 7 labeled PVN neurons from 3 rats (*p* > 0.05, Figure 6).

**FIGURE 7:**
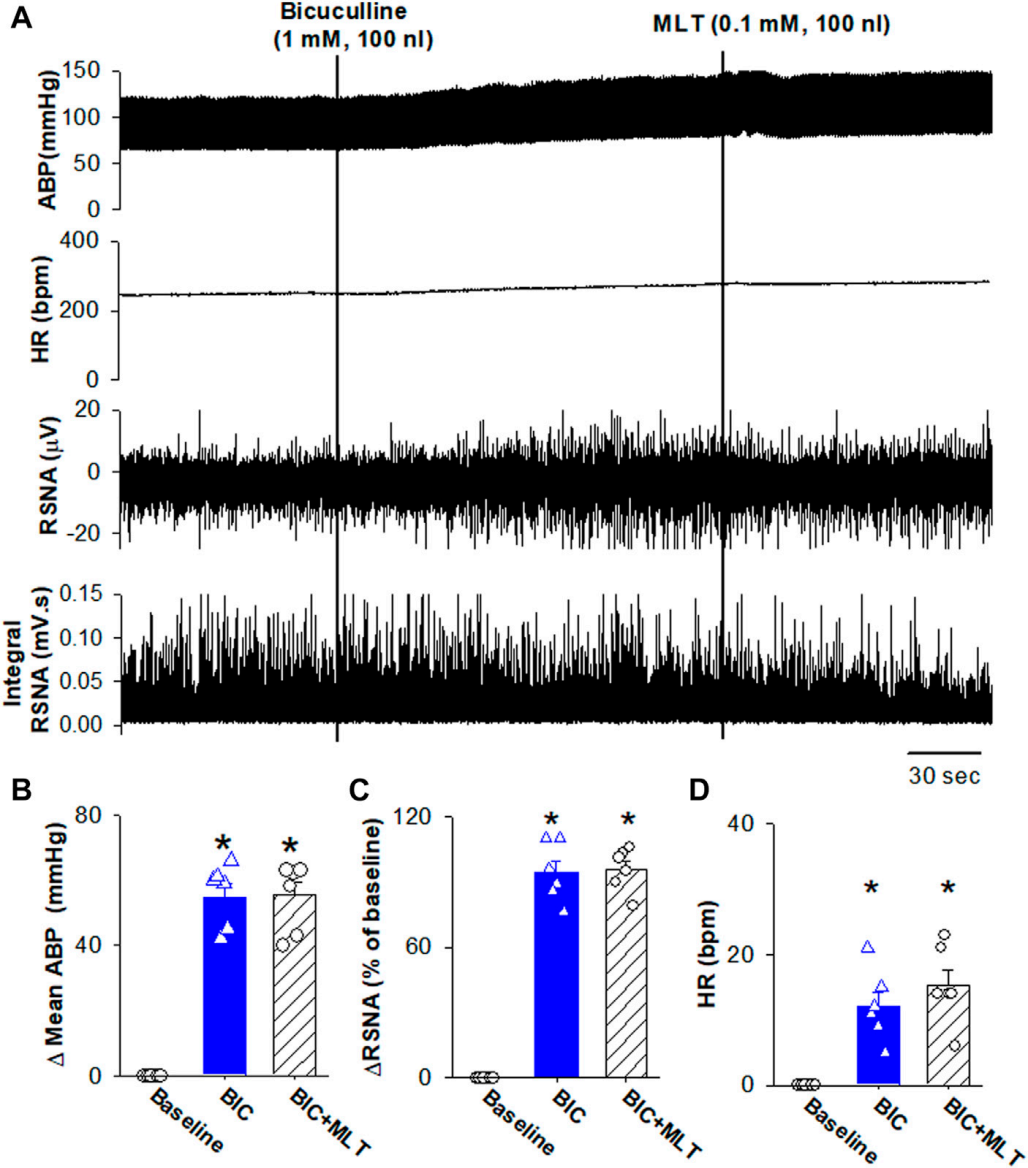
Blocking GABAA receptors in the PVN eliminated melatonin-induced decrease in blood pressure and sympathetic outflow. **(A)**, Representative raw traces show the effect of microinjection of bicuculline into the PVN increased ABP, RSNA, and HR. Subsequent microinjection of melatonin into the PVN did not significantly alter the ABP, RSNA, and HR. **(B–D)**, Summary data show changes in mean ABP **(B)**, RSNA **(C)**, and HR **(D)** in response to microinjection of bicuculine and melatonin into the PVN (n = 6). Data are presented as means ± SEM (*p < 0.05 compared with the control; ANOVA, followed by Dunn’s *post hoc* test). MLT, melatonin.

Because the RVLM projecting PVN neurons importantly drive sympathetic outflow (Jansen et al., 1995; Pyner and Coote, 2000; Coote, 2005), and GABA_A_ receptors mediated melatonin-induced decrease in firing activity of RVLM projecting PVN neurons, we determine if melatonin induces depressor response and sympathoinhibition through enhancing GABA_A_ receptors. Microinjection of bicuculline increased mean ABP from 85.3 ± 2.1 to 140.2 ± 3.7 mmHg (*p* < 0.0001, F_(2,15)_ = 173.6, n = 6 rats), RSNA from 100% to 194.5% ± 5.1% (*p* < 0.0001, F_(2,15)_ = 345.6, n = 6 rats), and HR from 293 ± 7.4 to 305.1 ± 7.1 bpm (*p* = 0.0003, F_(2,15)_ = 28.98, n = 6 rats, Figure 7). Subsequent microinjection of melatonin (1.0 mM, 100 nL) did not significantly alter the mean ABP, RSNA, and HR (*p* > 0.05, Figure 7). These data suggest that melatonin decreases ABP and sympathetic outflow through enhancing GABA_A_ receptor activity.

## 4 Discussion

This is the first study examining the cellular mechanisms involved in the effect of melatonin in the PVN on the presympathetic neurons and sympathetic vasomotor tone. We found that microinjection of melatonin into the PVN decreased arterial blood pressure and sympathetic outflow. The melatonin-induced depressor response and sympathoinhibition were not mediated by melatonin receptors. Furthermore, melatonin inhibited the firing activity of the retrogradely labeled RVLM projecting PVN neurons, an effect was eliminated by blocking GABA_A_ receptors with bicuculline, rather that melatonin receptor antagonist luzindole. In addition, melatonin enhanced the amplitude of evoked GABAergic IPSCs, GABA currents, and miniature IPSCs recorded from RVLM projecting PVN neurons. These data provide strong evidence that melatonin in the PVN suppresses sympathetic vasomotor tone through enhancing GABA_A_ receptor activity. Previous studies have indicated that Melatonin decreases blood pressure and sympathoadrenal tone (Laflamme et al., 1998; Scheer et al., 2003). It is expected that melatonin suppresses sympathetic nerve activity decrease blood pressure in hypertension rats. However, it is not clear if melatonin induces the same degree of suppressing sympathetic nerve activity in hypertensive rats.

Melatonin has pleiotropic functions in mammalian, brain through its different receptors, which are distributed in many brain regions including the PVN (Ng et al., 2017). However, we found that melatonin-induced depressor response and inhibition of sympathetic outflow and firing activity of PVN presympathetic neurons were not mediated by melatonin receptors since melatonin receptor antagonist luzindole did not attenuated the effect of melatonin on blood pressure, sympathetic outflow, and firing activity of PVN presympathetic neurons. In addition, luzindole did not alter the baseline blood pressure, sympathetic nerve activity, heart rate, as well as firing activity of PVN presympathetic neurons. These presympatehtic neurons provide critical excitatory drive to sympathetic outflow (Cano et al., 2001; Cano et al., 2004; Li et al., 2017). Previous studies have shown the melatonin act through mechanisms independent of specific receptors. For example, melatonin exerts antioxidation through scavenge hydroxyl (Bromme et al., 2000), superoxide (Sewerynek et al., 1996), peroxyl (Pieri et al., 1994), and NO free radical. (Noda et al., 1999). Melatonin-induced anti-oxidant properties may contribute to reduction of ischemia-reperfusion injury in various organs, including the heart (Tan et al., 1998a), kidney (Sahna et al., 2005) brain (Cho et al., 1997; Tan et al., 1998b). It has been shown that melatonin increases NO synthesis in the certain brain regions (Klimentova et al., 2016). In addition, melatonin directly binds to GABA_A_ receptors and induces a subsequent activation of GABA-receptors (Coloma and Niles, 1988; Niles, 1991; Wan et al., 1999; Li et al., 2001).

We found in this study that melatonin increased the amplitude of evoked GABAergic IPSCs. The increased amplitude of evoked GABAergic IPSCs was not attributes to increased GABA release, because melatonin did not alter the frequency of miniature GABAergic IPSCs, which reflect the presynaptic release probability. The evoked IPSCs was blocked by blocking GABA_A_ receptors with bicuculline, suggesting that melatonin enhance GABA_A_ receptor activity. This notion was strongly supported by additional findings that melatonin increased the amplitude of GABA_A_ receptor currents induced by puff application of GABA. Furthermore, melatonin also increased the amplitude of miniature IPSCs, suggesting melatonin enhanced the postsynaptic GABA_A_ receptor activity. Our findings are supported by previous reported data. For instance, melatonin enhances GABA binding in the rat brain (Coloma and Niles, 1988; Niles, 1991) and induce a similar maximal enhancement of GABA_A_ receptors as diazepam, which binds and enhance GABA_A_ receptor activity (Niles, 1991). Melatonin directly potentiates GABA-evoked current amplitude in SCN neurons (Wan et al., 1999), an effect were recapitulated by using heterologously expressed GABA_A_ receptors co-expressed with either MT1 or MT2 receptors (Wan et al., 1999). Similarly, melatonin was found to potentiate GABA_A_-evoked currents in cultured chick spinal cord neurons (Wu et al., 1999). Since melatonin increases current amplitude indued by exogenous application of GABA to native neurons, this action is due to an effect on GABA_A_ receptors rather than presynaptic effects. Consistently we also found that melatonin increased the amplitude of miniature IPSCs without affecting the frequency of miniature IPSCs. It has been proposed that melatonin acts as a positive allosteric modulator of GABA_A_ receptors since melatonin accelerates the decay time of GABA-evoked currents in chicken spinal cord neurons and carp retinal neurons (Wu et al., 1999; Li et al., 2001), an effect was not blocked by the melatonin receptor antagonist luzindole (Li et al., 2001), suggesting that melatonin act as an allosteric modulator of GABA_A_ receptor.

It has been shown that GABAergic signals in the PVN is enhanced by NO through increasing synaptic GABA release. In this regard, NO formation was shown to potentiate GABAergic inhibitory effects in PVN (Zhang and Patel, 1998; Li et al., 2002; Li et al., 2003; Rossi et al., 2004). Physiological concentrations of melatonin inhibit NO synthase activity in the hypothalamus (Bettahi et al., 1996; Leon et al., 2006; Fan et al., 2018). It is speculated that melatonin decreases GABA synaptic release through inhibition of NO synthesis. However, we found that melatonin did not alter the frequency of miniature GABAergic IPSC. These data suggest that NO-GABA interaction play minor role in the effect of melatonin on the regulation of PVN neurons and sympathetic vasomotor tone.

Since melatonin enhanced GABA_A_ receptor activity to inhibit the activity of PVN presympathetic neurons, while PVN presympathetic neurons are critical in regulating sympathetic outflow and blood pressure (Pyner and Coote, 2000; Cano et al., 2001; Li et al., 2017), in this study, we further assessed if melatonin-induced sympathoinhibition was mediated by GABA_A_ receptors. Consistently with previous studies, blocking GABA_A_ receptors with microinjection of bicuculline into the PVN increased blood pressure and sympathetic outflow (Zhang and Patel, 1998; Chen and Toney, 2003). Subsequent injection of melatonin did not change blood pressure and sympathetic outflow. Bicuculline induced an 100% increase in RSNA, while melatonin only produces 20% decrease in RSNA. Because melatonin did not induce any decrease in RSNA during bicuculline-induced sympathoexcitation, the lack of melatonin-induced sympathoinhibition is likely due to blockade of GABA_A_ receptor rather than bicuculline-induced sympathoexcitation. These data suggest that melatonin-induced sympathoinhibition was mediated by GABA_A_ receptors.

## 5 Conclusion

In this study, we found that melatonin inhibited firing activity of PVN presympathetic neurons, decreased blood pressure and sympathetic outflow. These effects are mediated by enhancing GABA_A_ receptor activity rather than through melatonin receptors. This study provides novel mechanisms underlying the effect of melatonin in the PVN on sympathetic outflow and blood pressure.

## Data Availability

The original contributions presented in the study are included in the article/Supplementary Material, further inquiries can be directed to the corresponding author.

## Abbreviations

ABP, arterial blood pressure; aCSF, artificial cerebrospinal fluid; HR, heart rate; LZD, luzindole; MLT, melatonin; PVN, paraventricular nucleus; RSNA, renal sympathetic nerve activity; RVLM, rostral ventrolateral medulla.
